# The real experiences of nurses after patient suicide: A meta-synthesis of qualitative studies

**DOI:** 10.1097/MD.0000000000040034

**Published:** 2024-10-25

**Authors:** Mengying Yang, Peipei Yu, Hui Zhang, Yingying Zheng

**Affiliations:** a Nursing Department, The Central Hospital of Wuhan, Tongji Medical College, Huazhong University of Science and Technology, Wuhan, China; b Cardiovascular Department, The Central Hospital of Wuhan, Tongji Medical College, Huazhong University of Science and Technology, Wuhan, China.

**Keywords:** meta-synthesis, nurses, patients suicide, qualitative research

## Abstract

**Objectives:**

To systematically evaluate the qualitative study of nurses’ real experience after patient suicide, and to provide theoretical basis for reducing the influence of patient suicide on nurses and reducing the incidence of patient suicide.

**Design:**

A systematic review and meta-synthesis of qualitative studies.

**Methods:**

These databases including The Cochrane Library, CINAHL, PubMed, Embase, Web of Science, CMB, CNKI, Wanfang, and VIP were searched by computer to collect relate qualitative researches. The retrieval time was from database established to February 2024. Joanna Briggs Institute Qualitative Research Quality Evaluation Form was used to evaluate the literature quality and Meta-integration.

**Results:**

A total of 12 studies were included and 47 results were obtained, which were summarized into 8 new categories and 3 integrated results were obtained. Theme 1: impact on nurses themselves; Theme 2: the nurse’s thoughts and suggestions of patient suicide; Theme 3: coping strategy.

**Conclusions:**

Nursing managers should fully pay attention to nurses’ feelings and cognitive attitudes after patients’ suicide events, strengthen psychological counseling for nurses, so as to reduce the impact of patients’ suicide events on nurses.

## 1. Introduction

Suicide is the behavior of an individual who intentionally or voluntarily uses various means to end his or her life, and is a very serious social and public health problem. Suicidal events do not only occur in the general population, but also in hospitalized patients who engage in suicidal behavior or make suicide attempts. According to the World Health Organization, more than 700,000 people die by suicide every year.^[1]^ The suicide death rate in China is 9.7/100,000,^[2]^ and compared with the general population, the suicide rate of inpatient is higher, which is 4 to 5 times that of the general population.^[3]^ Studies have shown that inpatient suicide not only brings great emotional trauma to the patient’s family, but also brings obvious trauma and impact to nurses as one of the personnel with whom the patient have the closest contact in the hospital.^[4]^ The psychological impact of patient suicide on nurses has received a great deal of attention in recent years. The majority of existing studies have adopted a qualitative methodology, yet to our comprehensive understanding, there exists a void in qualitative systematic reviews specifically addressing the psychological journey of nurses who have encountered inpatient suicide. Consequently, there arises a pressing need to gain a deeper appreciation of the psychological implications for nurses across diverse populations, spanning geographical and cultural landscapes, who have undergone this traumatic experience. In this paper, through integrating qualitative research on the real experiences of nurses after experiencing patient suicide events at home and abroad, we provide a comprehensive interpretation of the experience of nurses after experiencing patient suicide events, and understand their psychological experiences, attitudes, and perceptions, with a view for providing theoretical basis for improving the impact of patient suicide events on nurses and decreasing the incidence of patient suicide events.

## 2. Methods

Ethical approval was not required for this paper. The protocol for this study has been registered in PROSPERO, registration number: CRD42024557860.

### 2.1. Criteria for inclusion and exclusion

Our inclusion criteria: (1) the study subjects P (Population) were clinical nurses, aged ≥ 18 years, working registered nurses with some reading comprehension skills. (2) I (Interest of phenomena) is the nurse’s cognition, attitude, emotional experience, feelings, and experience formed after experiencing the patient’s suicide event.(3) Co (Context) nurses experience patient suicide events (including suicide ideas, suicide attempts and completed suicides).(4) S (Study design) is qualitative research, that is, a research method that uses a systematic, subjective approach to describe the lived experience and give meaning to it, including phenomenology, rooted theory, narrative research, ethnography, action research, and other qualitative research methods of the article.

Our exclusion criteria: (1) literature that is not available in full text; (2) literature in languages other than Chinese and English; (3) repeatedly published literature; (4) quantitative research.

### 2.2. Literature search strategy

The following databases were searched: The Cochrane Library, CINAHL, PubMed, Embase, Web of Science, and china biomedical literature database, CNKI, Wanfang database, and VIP database for relevant qualitative studies, and relevant references were traced, all from the time of construction to February 2024. A systematic search strategy was conducted by combining subject words and free words. The search strategy is provided in Appendix S1, Supplemental Digital Content, http://links.lww.com/MD/N700.

### 2.3. Literature screening and data extraction

Literature screening and data extraction was carried out independently by 2 researchers, discussion or consultation with a third researcher if there is disagreement between 2 researchers. The literature was screened by first de-duplicating the retrieved literature using NoteExpress programme, then by reading the titles and abstracts for initial screening, and then by reading the full text for re-screening to determine the final included literature. Data extraction included: authors, year of publication, country, research methodology, study population, and findings.

### 2.4. Literature quality appraisal

The included literature was evaluated independently by 2 researchers based on the Australian Joanna Briggs Institute Centre for Evidence-Based Health Care quality evaluation criteria for qualitative research. In case of disagreement between the 2 researchers, a discussion was held or a third investigator was consulted to assist in the judgement. The quality grade of all 12 studies included in this study was B.

### 2.5. Meta-synthesis

In this study, the results were integrated using the pooled integration method, which focuses on the nature and connotations of qualitative research and emphasizes the aggravation and function of qualitative research for evidence-based healthcare services.^[5]^ With a full understanding of the philosophical perspectives and research methods of qualitative research, the researcher repeatedly read the results of the study and their connotations, and analyzed the results, summarized the similar results to form new categories, and finally analyzed and summarized the new categories again as integrated results. The Preferred Reporting Items for Systematic Reviews and Meta-Analyses (PRISMA) 2020 checklist was used to report the meta-synthesis (see Appendix S2, Supplemental Digital Content, http://links.lww.com/MD/N701).

## 3. Results

### 3.1. Literature search results

**Figure 1.**
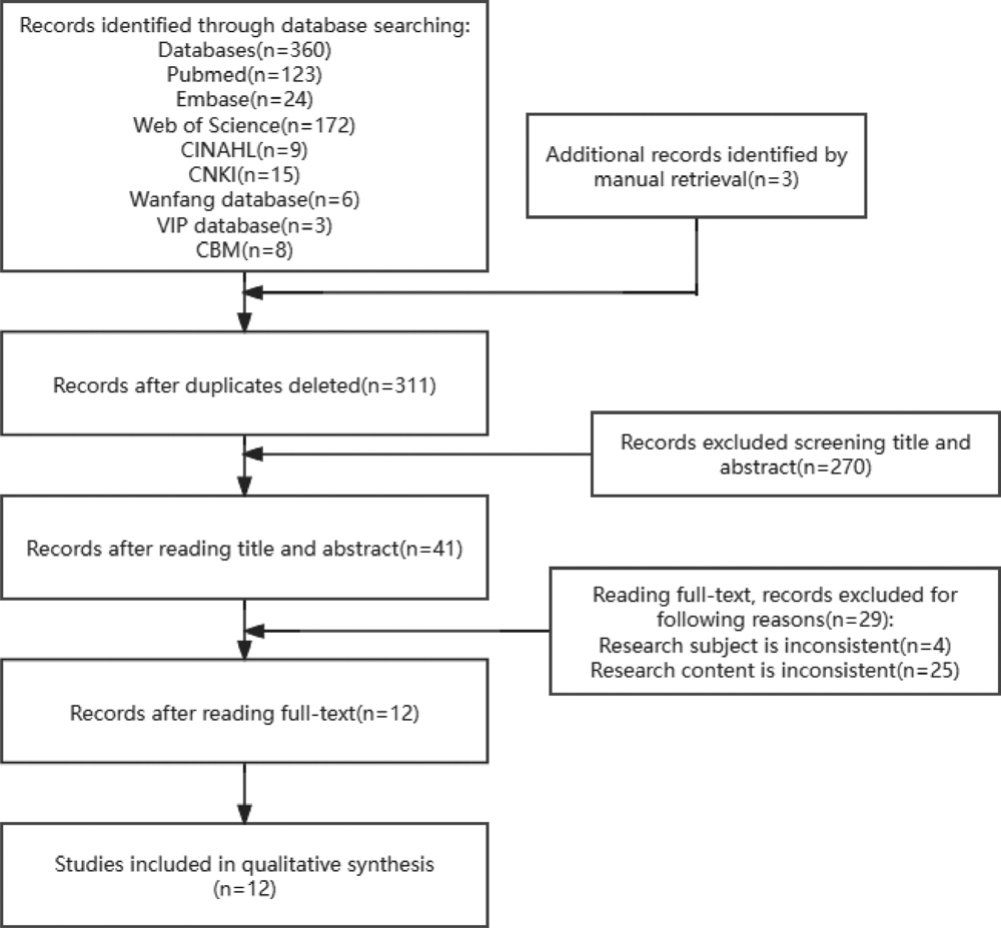
Flow diagram of literature screening.

According to the search strategy 363 articles were initially obtained and 12 articles were finally included, the literature screening process and results are shown in Figure 1.

### 3.2. Basic characteristics and quality assessment of the included literature

Twelve studies from different countries and cultures were included in this study, China (n = 8), South Africa (n = 1), Turkey (n = 1), Saudi Arabia (n = 1), and Ireland (n = 1), and the basic characteristics of the included studies are shown in Table 1. The quality grade of the 12 studies included in the present study was B, and the specific results are shown in Table 2.

**Table 1:** Characteristics of the literature.

Author/year	Country	Research design	Data collection	Study subjects	Hospital/Department	Results
Liu^[6]^/2020	China	Qualitative study	Semi-structured interview	Nurses who experienced inpatient suicide in the last 3 years volunteered to participate in the study (n = 15, 2 male and 13 female; age: 24–53)	General hospital	Three themes: afraid, terrified, and guilty
Yang^[7]^/2019	China	Phenomenological research	Semi-structured and individual in-depth interview	Oncology nurses who experienced patient suicide (n = 10, 1 male and 9 female; age: 22–36)	Oncology department	Four themes: shock, downcast, anxiety, depression
Wang^[8]^/2019	China	Phenomenological research	Semi-structured interview	Nurses who experienced patient suicide in the last 3 years volunteered to participate in the study and had normal language expression (n = 18, 2 male and 16 female; age: 26–50)	Hematology department	Three themes: positive attitude, ambivalence or neutrality, negative attitude towards patients suicide
Yang^[9]^/2019	China	Phenomenological research	Semi-structured interview	Nurses who experienced adverse events of patient suicide (n = 8)	Oncology hospital, Department of palliative care	Four themes: anxiety and fear, guilty and repentant, feeling of pressure, and inner pain
Zheng^[10]^/2019	China	Phenomenological research	Semi-structured and in-depth interview	Nurses who experienced inpatient suicide volunteered to participate in the study, had normal language expression and stable emotion(n = 7, 7 female; age: 26–33)	Internal medicine department	Five themes: negative emotional experience, greater psychological pressure, lack of nursing human resources, changes in professional identity, and individualized coping styles
Hu^[11]^/2014	China	Phenomenological research	Semi-structured and individual in-depth interview	Duty nurses who experienced patient suicide (n = 15, 1 male and 14 female; age: 23–38)	General hospital	Three themes: stressful and fear, self-blame and guilty, feeling of stress
Liu^[12]^/2010	China	Phenomenological research	Semi-structured interview	Nurses who experienced a patient suicide during their shift (n = 6, 6 female; age: 23–36)	General hospital, non-psychiatry department	Three themes: cognitive experience, affective experience, volitional experience
Matandela^[13]^/2017	South Africa	A qualitative design following interpretivism	individual in-depth interview	Nurses who experienced patient suicide (n = 6)	General hospital	Five themes: Experience of disbelief and helplessness, feelings of blame and condemnation, feelings of guilt and inadequacy, emotional reaction, fear of reprisal
Turkles^[14]^/2018	Turkey	Qualitative study	Individual in-depth interview	Nurses who experienced patient suicide volunteered to participate in the study(n = 33, age: 24–41,n = 21, age: 42 and over, n = 12)	Mental health and diseases hospital	Four themes: the nurses’ perspectives on the reasons why a patient may commit suicide, nurses’ feelings towards patients committing suicide, nurses’ interventions after a patient’s suicide attempt, nurses’ strategies for the prevention of suicide and the recommendations of nurses for suicide prevention
Alhamidi^[15]^/2020	Saudi Arabia	Constructivist phenomenological qualitative inquiry	Focus group	Practicing mental health nurses(n = 20, 3 male and 17 female)	Nursing College of King Saud University	Five themes: nurse experience of patient suicide, expressed feelings, psychological responses, the effect on practice, and support
Fiona^[16]^/2008	Ireland	Qualitative descriptive study	Semi-structured interview	Nurses working in acute psychiatric unit who experienced patient suicide or attempted suicide in the last 3 years(n = 9)	Acute psychiatric unit	Four themes: nurses’ experience of patient suicide/suicide attempts, nursing care following an incident of suicide/suicide attempt, feelings experienced by nurses following a suicide/suicide attempt and the support for nurses following a suicide/suicide attempt
Wang^[17]^/2016	China	Qualitative study	Semi-structured and individual in-depth interview	Clinical nurses who experienced inpatient suicide in the past 2 years(n = 15, 1 male and 14 female, age: 25–39)	Tertiary referral hospital	Four themes: nurses’ cognition about inpatient suicide, psychological reaction, impact on practice, patterns of regulation

**Table 2:** Methodological quality appraisal of the included studies.

Study	①	②	③	④	⑤	⑥	⑦	⑧	⑨	⑩	Quality level
Liu	U	N	N	U	U	N	N	Y	U	Y	B
Yang	Y	Y	Y	Y	Y	N	N	Y	U	Y	B
Wang	Y	Y	Y	Y	Y	N	N	Y	U	Y	B
Yang	Y	Y	Y	Y	Y	N	N	Y	U	Y	B
Zheng	Y	Y	Y	Y	Y	N	N	Y	Y	Y	B
Hu	Y	Y	Y	Y	Y	N	N	Y	N	Y	B
Liu	Y	Y	Y	Y	Y	N	N	Y	U	Y	B
Matandela	Y	Y	Y	Y	Y	N	N	Y	Y	Y	B
Turkles	Y	Y	Y	Y	Y	N	N	Y	Y	Y	B
Alhamid	Y	Y	Y	Y	Y	N	N	Y	Y	Y	B
Fiona	Y	Y	Y	Y	Y	N	N	Y	Y	Y	B
Wang	Y	Y	Y	Y	Y	N	N	Y	Y	Y	B

*Note*: Appraisal Checklist: ① Is there congruity between the stated philosophical perspective and the research methodology? ② Is there congruity between the research methodology and the research questions or objectives? ③ Is there congruity between the research methodology and the data collection methods? ④ Is there congruity between the research methodology and the representation and the data analysis? ⑤ Is there congruity between the research methodology and the interpretation of results? ⑥ Is there a statement locating the researcher culturally or theoretically? ⑦ Is the influence of the researcher on the research, and vice- versa, addressed? ⑧ Are participants and their voices adequately represented? ⑨ Is the research ethical according to current criteria or, for recent studies, and is there evidence of ethical approval by an appropriate body? ⑩ Do the conclusions drawn from the research report flow from the analysis or interpretation of the data?

Appraisal result: Y = yes; N = no; U = unclear; N/A = not applicable.

### 3.3. Meta-synthesis

Through repeated reading, comprehension, analysis, and interpretation of the 12 included studies, the researchers extracted 47 clear research results, summarized and combined similar results into 8 new categories, and then synthesized them into 3 themes (see Appendix S3, Supplemental Digital Content, http://links.lww.com/MD/N702).

#### 3.3.1. Theme 1: Impact on nurses themselves

**Category 1: Emotional experiences after patient suicide**

Most nurses initially express shocked and disbelief when they discover a patient has committed suicide. (“The patient flew down like a bird. It was so shocking. I didn’t even want to look down at that patient, I was shocked and shivering”^[13]^).

Patients suicide have a strong internal impact on nurses, who feel scared and intimidated. (“I’m afraid. I’m afraid of going through the same thing at any time. I always check on my patients at night. I check to see if they’re dead or their eyes are open, if they’re breathing. I always check the restrooms. I’m afraid whenever I am on duty, I wonder if someone has cut him/herself or drunk something”;^[14]^ “I’m terrified of remembering when a patient dies by suicide.”^[11]^)

The nurses feel guilty for failing to find the patient’s suicide intention in time, failing to prevent the patient from committing suicide in time in their daily work. (“I felt that, because of my inability to save my patient, I had failed their families, who had entrusted me with the care of their loved ones”;^[15]^ “The extent to which I blamed myself affected me deeply. I could not sleep and felt responsible for the patient’s death, as though in some way I contributed to make it happen. I failed the patient due to inadequate nighttime observation”;^[15]^ “This incident left me traumatized and depressed, and I was prescribed antidepressants which I still require.”^[15]^).

**Category 2: Fear of blame**

Nurses feel sorry to higher-ups after experiencing patients suicide. Nurses are fear of blame from leaders and loss of job. (“I’m afraid of being blamed by the leadership”;^[12]^ “Nurses are blamed as always. Why did you neglect the patient, why did you not look after, why this, why that. If the patient dies, the investigation begins. There is little other than a direct questioning of the nurse’s fault. Nobody says that he committed suicide because he was sick. The nurse’s fault is directly questioned”;^[14]^ “I’m afraid I’ll be removed from the South African Nursing Council”^[13]^).

Nurses fear that the family will not be able to accept the patient’s suicide and the loss of their loved one, blaming the nurse and fearing condemnation and threats from the family. Meanwhile nurses are worried about medical disputes. (“I remember family members who pointed their fingers at me and said I promise you’ll never work again’”;^[13]^ “The family members said you’re responsible for my brother’s death because you caused him to jump out of a window instead of being taken care of”^[13]^).

**Category 3: Impact on practice and professional identity**

Nurses believe that busy clinical workloads and high occupational risks put a lot of psychological pressure on nurses. Constant job puts nurses in a state of stress, which leads to burnout. (“It’s not a good job. Not only are you busy, but you have to deal with patients committing suicide, so you’re always on edge”;^[10]^ “I felt like there was a stone on my chest which makes me breath hard, I doubt weather I could do this job anymore”;^[17]^ “The scene of the patient collapsed in a pool of blood, life is really so fragile, I can’t love this profession”;^[10]^ “The thought of changing careers is getting stronger ”^[10]^).

#### 3.3.2. Theme 2: The nurse’s thoughts and suggestions of patient suicide

**Category 4: Attitudes towards patient suicide**

Nurses who experienced patient suicide have an understanding and sympathetic attitude towards patient suicide and believe that patient’s choice should be respected, it’s also a relief for the patients (“It’s a painful thing that a person doesn’t even have the right to die, and we should respect his choice and wishes”;^[8]^ “Maybe this patient really can’t stand it anymore, both physically and psychologically, and feels like he’s worse than dead”;^[8]^ “This patient was so tired at the end of the day, it was painful for me to watch. It was a relief.”^[8]^).

Nurses who experienced patient suicide are ambivalent or neutral towards patients suicide; nurses understand the patient’s choice to commit suicide but do not support patient suicide due to professional ethics and morals (“I can understand a patient committing suicide, but I can’t support it for professional, ethical reasons”^[8]^). Some nurses express understanding that patients should be allowed to leave with dignity, rather than choosing to go to extremes (“Suicide may be a kind of relief for the patient himself, and his choice should be respected, but we should minimize his pain and let the patient leave with dignity, instead of choosing such an extreme way to end his own life.”^[8]^).

Nurses who experienced patient suicide are opposed to and do not understand patient suicide, and nurses believe that patients take suicidal behavior without considering the feelings of their families (“I think the person who committed suicide was very narrow minded and didn’t think about how his parents and children would have to deal with the outcome.”^[8]^). It is not considered advisable for patients to choose suicide (“When you are suddenly under a lot of pressure, your first reaction is to run away, or in the extreme case, to commit suicide, which is very undesirable.”^[8]^).

**Category 5: The nurse’s thoughts on the cause of patient suicide**

Owing to the lack of human resources for nursing, especially in developing countries, nurses do not have enough time to conduct ward rounds and communicate with patients to detect suicidal intentions and prevent suicides in a timely manner, especially at night, when there are fewer nurses and their workload is heavy (“I didn’t notice any abnormalities in the patients when I took over the shift that night, and with a particularly high number of treatments and infusions scheduled, I was so busy with the treatments and infusions that I didn’t have time to make ward rounds in a timely manner”;^[10]^ “Our wards have a capacity of 8 patients and we are not able to observe these patients on a one-to-one basis in the wards”^[14]^).

Nurses are not sufficiently aware of patients suicide prevention and never think of patients committing suicide. The belief that patients suicide is unavoidable and that is an accident that cannot be prevented (“I’ve never had an inpatient suicide, I don’t think it is something I will experience”;^[15]^ “We can’t give them 24-h surveillance, if patient had determined, he would commit suicide out of our sight. It is really inevitable in certain situation, no matter how hard we try.”^[15,17]^).

Nurses feel unable to provide appropriate psychological support to patients with suicidal intent. In some cases, when patients have suicidal thoughts or intentions, but due to lack of training, nurses do not intervene effectively with the patient (“We have limited skills in suicide prevention”;^[15]^ “The subject about death is such a taboo for patients. I won’t ask them ‘Do you have the suicide ideation’ directly, and I don’t know how to ask or detect their suicide ideation, how to comfort. Sometimes words are powerless”;^[17]^ “If a patient is depressed, we do not automatically associate them with suicide. I feel that I lack the necessary experience and skills to detect potential suicide, and I need to be trained to acquire them”^[15]^).

**Category 6: The nurse’s suggestions for preventing patient suicide**

Checking wards for objects with sharp points or edges, such as knives, forks and scissors, keeping substances such as iodine and alcohol out of the reach of patients, and preventing patients from carrying any medication, scarves, belts or shoelaces. Nurses also suggest the use of cameras to monitor patients, and due to the busy schedules of nurses, the use of cameras assisted nurses in observing patients to prevent them from committing suicide (“Shoelaces and ropes should be taken away so patients don’t hurt or hang themselves, and for women, scarves should be taken away as well”;^[14]^ “Our wards have a capacity of 8 patients and we are not able to observe these patients on a one-to-one basis in the wards”^[14]^).

In order to prevent suicide among hospitalized patients, it is necessary to listen to patients, give them the opportunity to express their feelings, enhance communication with patients, and try to understand their inner needs. Giving patients appropriate emotional support to make them feel valued and cared for (“We should take care of our patients with love, we should love them and we should respect them; after all, they are individuals and they have the right to be loved and respected. I don’t think people who feel they love others and are loved by them commit suicide ”^[14]^).

#### 3.3.3. Theme 3: Coping strategy

**Category 7: Seeking support and pouring out emotion**

Nurses who experienced patients suicide should positively confront the trauma and seek psychological support, including talking to coworkers and family members, controlling emotions and emotional expression (“Overall, talking to colleagues on the ward provided me with the most support and we were able to share each other’s experiences and feelings”;^[15]^ “The biggest help for me is interacting with my peers on the ward, and I think that is the biggest help. You know that you have support from other nurses who have similar experiences”;^[16]^ “I need comfort but the head nurse didn’t realize. My colleagues comforted me but did not work. I poured out my grievances to my parents because they found I was in low mood.”^[17]^).

**Category 8: Provision of suicide prevention education**

Proposals include the provision of specialized suicide-related education for nurses, strengthening the level of psychological care for nurses, and enhancing the knowledge and skills of nurses (“There would be real value in providing nurses with professional education about suicide, in particular how to respond appropriately to suicide, and in better understanding the impact of suicide on staff and patients’ family.”^[15]^).

## 4. Discussion

### 4.1. Rational and optimal allocation of human resources

From the results of this Meta-integration, it was concluded that nurses do not have enough time at night to make ward rounds as well as communicate with patients to detect suicidal intent in time, increasing the risk of suicidal events. Nursing managers should quantify nursing workload as specifically as possible and allocate nursing human resources reasonably,^[18]^ so as to avoid the occurrence of nursing staff taking care of too many patients and overloaded work, which leads to untimely patrol by nurses and increase the risk of patient suicidal events. The department should set up an additional mobile nurse pool in the department as a reserve, and make timely deployment of personnel in the pool according to the needs of the specific workload, so as to reasonably allocate nursing human resources and alleviate the pressure of nurses who are unable to effectively respond to the excessive workload.

### 4.2. Enhancing nurse’s knowledge of patient suicide prevention

From the results of this Meta-integration, it was concluded that nurses have inadequate knowledge about the prevention of suicide in patients. Studies have shown that nurses in general hospitals have a low level of suicidal cognition, and even have serious misconceptions about suicide.^[19–21]^ Nursing staffs have the closest contact with patients, and their knowledge of patient suicide prevention directly affects the ability to recognize and detect patient suicide. One of the most important measures to prevent patient suicide is the timely detection and identification of patients with suicidal intent. Studies have shown that suicide prevention education and training is one of the most effective means of preventing patient suicide.^[22,23]^ Therefore, it is very necessary to carry out education related to patient suicide prevention for nurses, so that nurses can realize that patient suicide can be prevented, and strengthen nurses’ understanding of patients’ abnormal behaviors before suicide, such as writing wills, being depressed, inquiring about the mode of death, quarreling with family members, and showing anorexic thoughts, etc, so that nurses will pay attention to patients’ abnormal behaviors, and actively take on the important responsibility of preventing patient suicide, and do a good job of the patient’s suicide’s gatekeeper role.^[24]^ Currently, suicide prevention training programmes for nurses tend to be brief, focusing on knowledge transfer in the form of lectures designed to provide knowledge about suicide prevention and to equip them to provide supportive help to patients at risk of suicide.^[25]^ However, for nurses who need to identify patients at risk of suicide and provide appropriate interventions, knowledge transfer in the form of lectures alone is not sufficient, and practical education to improve counseling skills and intensive courses supplemented by individual feedback are required. In contrast, active learning is an interactive process that builds competence through an iterative cycle of behavioral practice and review, and promotes the potential development and application of skills.^[26]^ Long-term training facilitates a variety of learning method strategies, such as group focus interviews, case study analysis sessions, scenario simulations, and interactive group discussions. In clinical practice, suicide prevention training models with counseling practices and through case presentations are also highly recognized by nurses as effective ways to achieve suicide prevention education.^[27]^ It can be seen that long-term training can provide nurses with more opportunities to practise their skills, and training and learning through various educational methods are conducive to the development of nurses’ ability to practice suicide prevention. Since the barriers to suicide risk and the characteristics of patients’ illnesses are different in different departments, studies have shown that the development of suicide prevention training programmes with different subgroups and levels of training for specific populations can achieve good results.^[28]^ Therefore, nursing administrators should target suicide prevention training to different subspecialty nurses based on the characteristics of patients’ diseases and patients’ suicide risk factors in their units.

### 4.3. Enhancing psychological interventions to improve nurse’s coping styles

From the results of this Meta-integration, it was concluded that after experiencing a patient suicide event, nurses develop a series of negative emotions, including shock, panic, fear, dread, helplessness, guilt, frustration, self-blame, and depression, which affects the normal work and life of the nurses, which is in line with the findings of Busch.^[29]^ The study showed that intervened with nurses experiencing patient suicide events by adopting the Balint group method, and the results showed that the Balint group method can effectively improve the negative emotions of patients, and the method enables nurses to express their own experiences and feelings, providing nurses with a cathartic and communicative environment, and the method is highly maneuverable, which is worthy of being popularized and applied in the clinic.^[30]^ This study found that nurses experiencing patient suicide adopt negative coping methods in the face of the negative event of patient suicide, and the study showed that negative coping methods are not conducive to physical and mental health,^[31]^ while positive coping methods can not only reduce the adverse effects of stress on the individual, but also improve the individual’s ability to cope with the external pressure, so nursing managers should guide nurses to positively cope with the event of patient suicide and correctly face the event of patient suicide.

## 5. Summary

This study used the meta-synthesis method of qualitative research to explore the real experience of nurses experiencing patient suicide events, which profoundly explained the attitudes, emotions, cognitions, and feelings of nurses after experiencing patient suicide events. The study suggests that in future studies, nursing managers are recommended to pay full attention to the real experiences of nurses after experiencing patient suicide events, to pay attention to the psychological changes of nurses, to alleviate the negative emotions of nurses, and, at the same time, to carry out suicide-related education and training for nurses to effectively prevent hospitalized patients from committing suicide.

## Author contributions

**Conceptualization:** Mengying Yang.

**Data curation:** Mengying Yang, Hui Zhang.

**Methodology:** Mengying Yang, Yingying Zheng.

**Resources:** Hui Zhang.

**Writing – original draft:** Mengying Yang.

**Writing – review & editing:** Peipei Yu.

## Supplementary Material


