# Supplementary material for: The real experiences of nurses after patient suicide: A meta-synthesis of qualitative studies
Source: Medicine (Baltimore). 2024 Oct 25;103(43):e40034. doi: 10.1097/MD.0000000000040034 (PMC11521019; doi:10.1097/MD.0000000000040034)
Supplement: Supplementary file 1 [file medi-103-e40034-s001.doc]

**Appendix S1**

Literature search strategy

| #1 Nurses[MeSH Terms] |
| --- |
| #2 Nurs*[Title/Abstract] OR Nursing Personnel[Title/Abstract] OR Personnel, Nursing[Title/Abstract] OR Registered Nurs*[Title/Abstract] OR Nurs*, Registered[Title/Abstract] |
| #3 #1 OR #2 |
| #4 Patients[MeSH Terms] |
| #5 Patient*[Title/Abstract] OR Client*[Title/Abstract] |
| #6 #4 OR #5 |
| #7 Suicide[MeSH Terms] |
| #8 Suicid*[Title/Abstract] |
| #9 #7 OR #8 |
| #10 Qualitative Research[MeSH Terms] |
| #11 Research, Qualitative[Title/Abstract] OR Qualitative Study[Title/Abstract] OR Qualitative Method[Title/Abstract] OR Phenomen*[Title/Abstract] OR  Grounded Theory[Title/Abstract] OR Narrative Research[Title/Abstract] |
| #12 #10 OR #11 |
| #13 #3 AND #6 AND #9 AND #12 |
