# Supplementary figures and images for: The real experiences of nurses after patient suicide: A meta-synthesis of qualitative studies
Source: Medicine (Baltimore). 2024 Oct 25;103(43):e40034. doi: 10.1097/MD.0000000000040034 (PMC11521019; doi:10.1097/MD.0000000000040034)

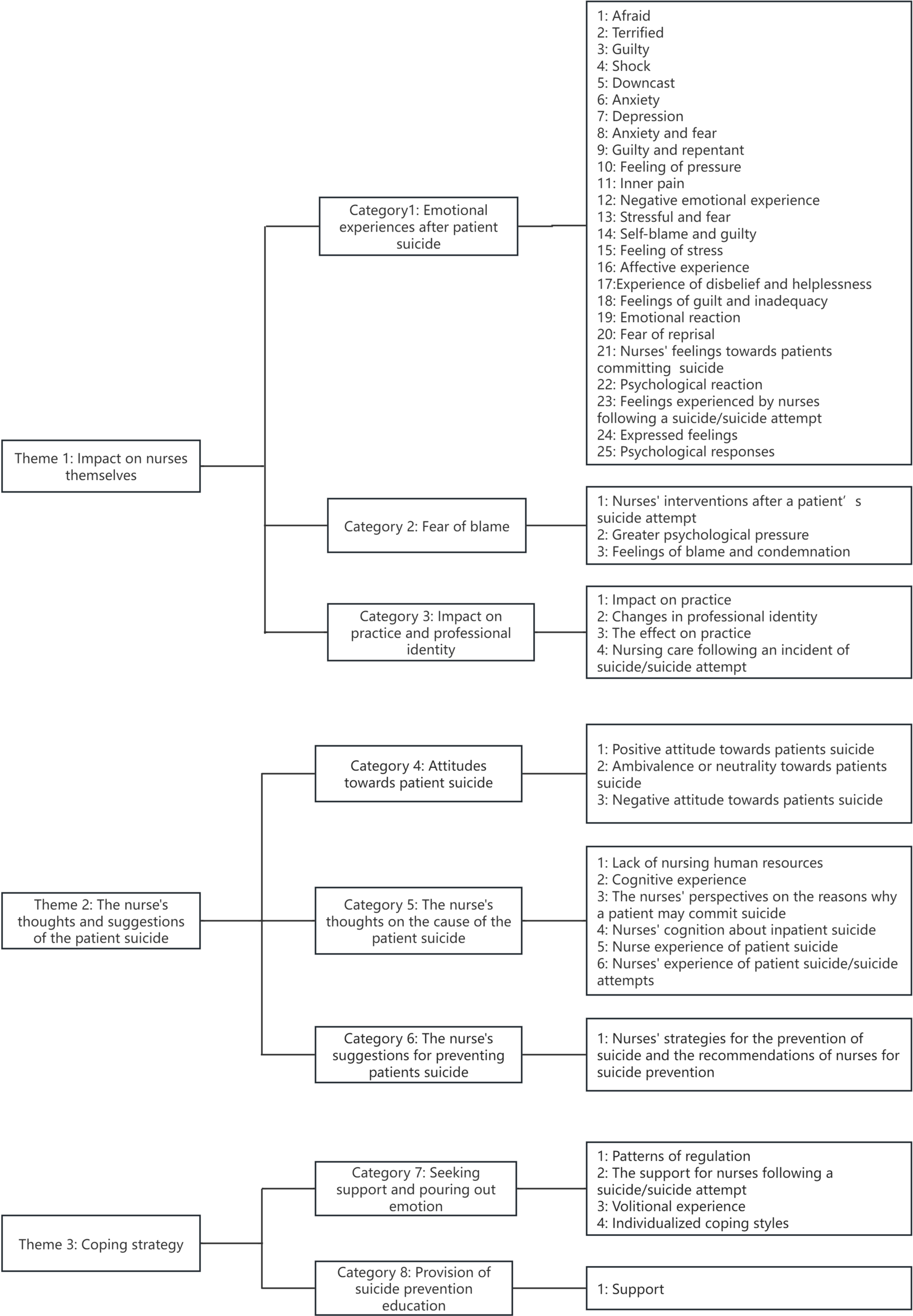

Supplement: Supplementary file 3 [file medi-103-e40034-s003.pdf]
